# Chronic L-Name-Treatment Produces Hypertension by Different Mechanisms in Peripheral Tissues and Brain: Role of Central eNOS

**DOI:** 10.3390/pathophysiology27010007

**Published:** 2020-12-15

**Authors:** Olga Pechanova, Stanislava Vrankova, Martina Cebova

**Affiliations:** Centre of Experimental Medicine, Slovak Academy of Sciences, Institute of Normal and Pathological Physiology, 813 71 Bratislava, Slovakia; Stanislava.Vrankova@savba.sk (S.V.); martina.cebova@savba.sk (M.C.)

**Keywords:** hypertension, heart, aorta, kidney, brain, nitric oxide, eNOS, L-NAME, NF-κB

## Abstract

The goal of our study was to analyze the time course of the effect of N^G^-nitro-L-arginine methyl ester (L-NAME) on nitric oxide synthase (NOS) isoforms and nuclear factor–κB (NF-κB) protein expression, total NOS activity, and blood pressure (BP) in rats. Adult 12-week-old male Wistar rats were subjected to treatment with L-NAME (40 mg/kg/day) for four and seven weeks. BP was increased after 4- and 7-week L-NAME treatments. NOS activity decreased after 4-week-L-NAME treatment; however, the 7-week treatment increased NOS activity in the aorta, heart, and kidney, while it markedly decreased NOS activity in the brainstem, cerebellum, and brain cortex. The 4-week-L-NAME treatment increased eNOS expression in the aorta, heart, and kidney and this increase was amplified after 7 weeks of treatment. In the brain regions, eNOS expression remained unchanged after 4-week L-NAME treatment and prolonged treatment led to a significant decrease of eNOS expression in these tissues. NF-κB expression increased in both peripheral and brain tissues after 4 weeks of treatment and prolongation of treatment decreased the expression in the aorta, heart, and kidney. In conclusion, decreased expression of eNOS in the brain regions after 7-week L-NAME treatment may be responsible for a remarkable decrease of NOS activity in these regions. Since the BP increase persisted after 7 weeks of L-NAME treatment, we hypothesize that central regulation of BP may contribute significantly to L-NAME-induced hypertension.

## 1. Introduction

Chronic inhibition of nitric oxide synthase (NOS) by N^G^-nitro-L-arginine methyl ester (L-NAME) is a well-established model of experimental hypertension and organ damage within the cardiovascular system [1,2,3] and kidney [4,5,6]. However, the mechanisms responsible for blood pressure (BP) increase following the organ changes in this type of hypertension have not been fully elucidated.

The mechanism of L-NAME-induced hypertension involves more than a simple inhibition of NO production with a consequent decrease of vasorelaxant activity. Nevertheless, attenuated vascular relaxation [7,8] and enhanced contraction in different parts of the vascular tree [9,10] are the first factors contributing to the increase of blood pressure. Among the other factors, increased activity of the renin–angiotensin–aldosterone system (RAAS) [11,12,13] and sympathetic nervous system (SNS) [14,15] were demonstrated by a number of authors. Increased production of prostaglandins [16] and reactive oxygen species (ROS) [17,18,19] were described as additional serious factors contributing to the development of L-NAME-induced hypertension.

Several studies documented induction of inducible NOS (iNOS) as a marker of the inflammation process following chronic L-NAME treatment [20,21,22]. Silambarasan et al. [23] and Berkban et al. [24] showed decreased endothelial (eNOS) gene expression after long-term L-NAME treatment, while Grumbach et al. [25] reported increased eNOS mRNA levels in endothelial cells after in vitro L-NAME administration. The studies are rather contradictory, since one could expect induction of eNOS expression after chronic L-NAME treatment as a consequence of the process of adapting to both decreased NOS activity and increased blood pressure. The mechanism of eNOS induction after L-NAME treatment, described by Grumbach et al. [25], includes activation of the transcriptional regulatory protein nuclear factor–κB (NF-κB), usually associated with iNOS induction [26,27]. Moreover, Kitamoto et al. [28] demonstrated that NF-κB participated in the L-NAME-induced alterations independently of the blood pressure increase.

The aim of our study was to elucidate the time-dependent effect of L-NAME on eNOS, neuronal NOS (nNOS), and iNOS and NF-κB protein expression in peripheral tissues, i.e., the aorta, heart, and kidney, and in brain regions, i.e., the brainstem, cerebellum, and brain cortex.

## 2. Materials and Methods

### 2.1. Chemicals and Drugs

Most chemicals used were purchased from Sigma Chemicals, Germany; if not, the company is indicated.

### 2.2. Animals and Treatment

All procedures and experimental protocols were approved by the Ethical Committee of the Institute of Normal and Pathological Physiology, SAS, and conform to the European Convention on Animal Protection and Guidelines on Research Animal Use.

Male Wistar rats, 12 weeks old, were divided into the control group (n = 16) and the group treated with N^G^-nitro-L-arginine methyl ester in the dose 40 mg/kg/day (n = 16). After four weeks of treatment, 8 randomly chosen rats from each group were sacrificed (groups: control 4 and L-NAME 4). The remaining 8 rats from the L-NAME-treated group received L-NAME in the same dose for the next three weeks (group: L-NAME 7) and the residual 8 rats from the control group served as age-matched controls (group: control 7). L-NAME was administered via the drinking water from the 12th week of age for 4 and 7 weeks. Daily water consumption was estimated individually for every animal one week before the experiment. During the experiment, drinking fluid consumption was controlled and adjusted, if necessary. All animals were housed at a temperature of 22–24 °C, in individual cages and fed with a regular pellet diet ad libitum.

Blood pressure (BP) was measured by the non-invasive method of tail-cuff-plethysmography every week. At the end of treatment, the animals were sacrificed, and body weight (BW) and left ventricle weight (LVW) were determined. The LVW/BW ratio was calculated. Total NOS activity, eNOS, nNOS, iNOS, and NF-κB (p65) protein expressions were determined in the aorta, heart, kidney, brainstem, cerebellum, and brain cortex.

### 2.3. Total NO Synthase Activity

Total NOS activity was determined in crude homogenates of the tissues by measuring the formation of [^3^H]-L-citrulline from [^3^H]-L-arginine as described previously [29].

### 2.4. Western Blot Analysis

Tissue samples of each investigated animal were homogenized in lysis buffer – 0.05 mM Tris containing protease inhibitor cocktail (Sigma-Aldrich, Germany). After centrifugation (15,000 rpm at 4 °C for 20 min), protein concentrations were determined by Lowry assay. Proteins were subjected to 10% SDS-PAGE and transferred onto nitrocellulose membrane. Membranes were blocked with 5% non-fat milk in Tris-buffer solution (TBS; pH 7.6) containing 0.1% Tween-20 (TBS-T) for 1 h at room temperature and then incubated in the presence of the appropriate primary antibodies overnight at 4 °C with polyclonal rabbit anti-endothelial NOS (1 μg/mL), anti-neuronal NOS (1 μg/mL), anti-inducible NOS (1 μg/mL) antibodies, anti-GAPDH (0.4 μg/mL) as a loading control (Alexis Biochemicals, Germany), and a polyclonal rabbit anti-nuclear factor-κB (NF-κB) antibody (0.5 μg/mL), which recognizes the 65-kDa RelA (p65) protein (Santa Cruz Biotechnology, CA, USA). Membranes were washed and finally incubated with secondary antibodies for 2 h at room temperature using a secondary peroxidase-conjugated anti-rabbit antibody (Alexis Biochemicals, Germany). After a final wash, chemiluminescence reagents (ECL, Amersham, Buckinghamshire, England) were used and the membrane exposed the X-ray film. The intensity of bands was analyzed using Photo-Capt V.99 software. The expression levels of e-NOS, nNOS, iNOS, and NF-κB were compared with that of the standardized GAPDH expression levels.

### 2.5. Statistical Analysis

The results are expressed as mean ± S.E.M. One-way analysis of variance and Bonferroni test were used for statistical analysis. Values were considered to differ significantly if the probability value was less than 0.05.

## 3. Results

### 3.1. Cardiovascular Parameters

**Figure 1:**
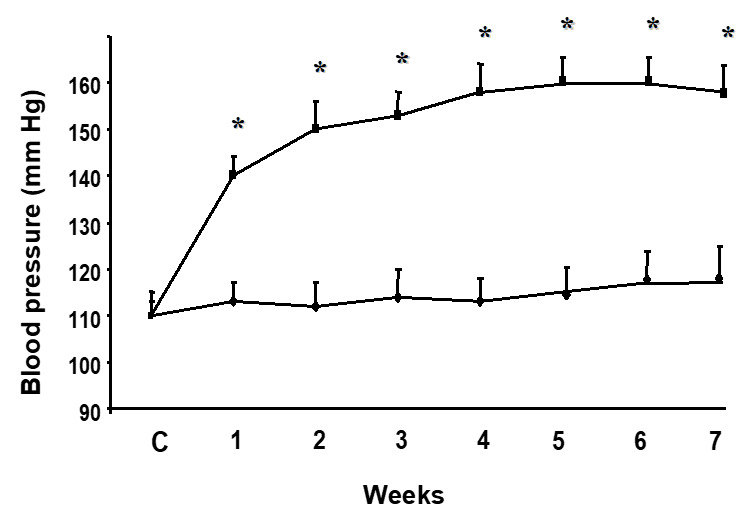
Effect of N^G^-nitro-L-arginine methyl ester (L-NAME) treatment on blood pressure (BP) increase. * *p* < 0.05 compared to age-matched controls.

Four-week L-NAME treatment increased BP in comparison with controls. Prolongation of the treatment to 7 weeks kept the blood pressure on the level of 4-week L-NAME-treated rats (Figure 1). BW was not significantly different among individual experimental groups (i.e., control 4, L-NAME 4, control 7, L-NAME 7). Both 4- and 7-week L-NAME treatments induced an increase in the LVW/BW ratio in comparison with age-matched control rats (Table 1).

### 3.2. Total NOS Activity

**Figure 2:**
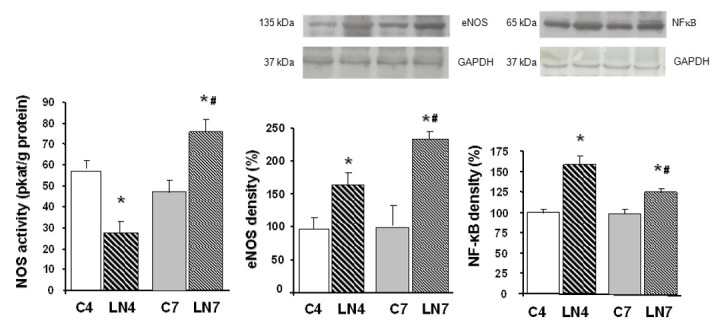
Effect of 4- and 7-week N^G^-nitro-L-arginine methyl ester (L-NAME) treatment on NO synthase (NOS) activity, endothelial NOS (eNOS) protein expression, and nuclear factor-kappaB (NF-κB, p65) protein expression in the aorta. Group treated with L-NAME for 4 weeks (LN4) and age-matched controls (C4), group treated with L-NAME for 7 weeks (LN7) and age-matched controls (C7). * *p* < 0.05 compared to age-matched controls, # *p* < 0.05 compared to LN4.

**Figure 3:**
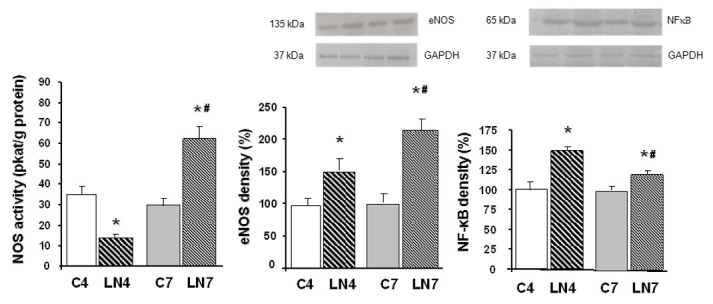
Effect of 4- and 7-week N^G^-nitro-L-arginine methyl ester (L-NAME) treatment on NO synthase (NOS) activity, endothelial NOS (eNOS) protein expression, and nuclear factor-kappaB (NF-κB, p65) protein expression in the heart. Group treated with L-NAME for 4 weeks (LN4) and age-matched controls (C4), group treated with L-NAME for 7 weeks (LN7) and age-matched controls (C7). * *p* < 0.05 compared to age-matched controls, # *p* < 0.05 compared to LN4.

**Figure 4:**
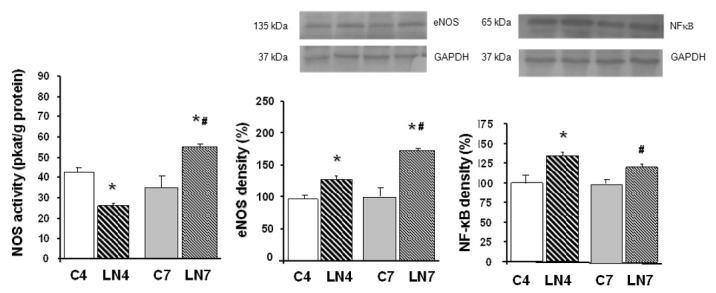
Effect of 4- and 7-week N^G^-nitro-L-arginine methyl ester (L-NAME) treatment on NO synthase (NOS) activity, endothelial NOS (eNOS) protein expression, and nuclear factor-kappaB (NF-κB, p65) protein expression in the kidney. Group treated with L-NAME for 4 weeks (LN4) and age-matched controls (C4), group treated with L-NAME for 7 weeks (LN7) and age-matched controls (C7). * *p* < 0.05 compared to age-matched controls, # *p* < 0.05 compared to LN4.

**Figure 5:**
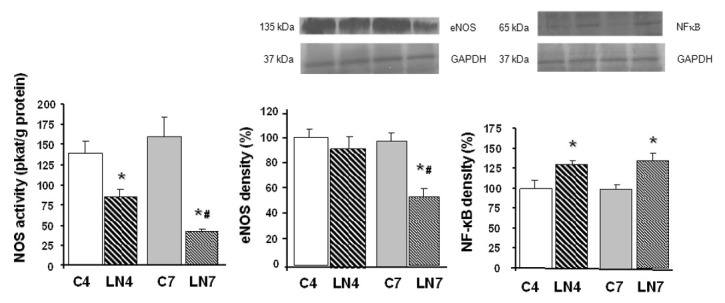
Effect of 4- and 7-week N^G^-nitro-L-arginine methyl ester (L-NAME) treatment on NO synthase (NOS) activity, endothelial NOS (eNOS) protein expression, and nuclear factor-kappaB (NF-κB, p65) protein expression in the brainstem. Group treated with L-NAME for 4 weeks (LN4) and age-matched controls (C4), group treated with L-NAME for 7 weeks (LN7) and age-matched controls (C7). * *p* < 0.05 compared to age-matched controls, # *p* < 0.05 compared to LN4.

**Figure 6:**
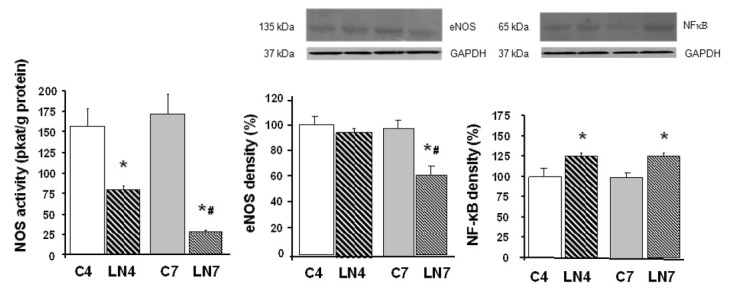
Effect of 4- and 7-week N^G^-nitro-L-arginine methyl ester (L-NAME) treatment on NO synthase (NOS) activity, endothelial NOS (eNOS) protein expression and nuclear factor-kappaB (NF-κB, p65) protein expression in the cerebellum. Group treated with L-NAME for 4 weeks (LN4) and age-matched controls (C4), group treated with L-NAME for 7 weeks (LN7) and age-matched controls (C7). * *p* < 0.05 compared to age-matched controls, # *p* < 0.05 compared to LN4.

**Figure 7:**
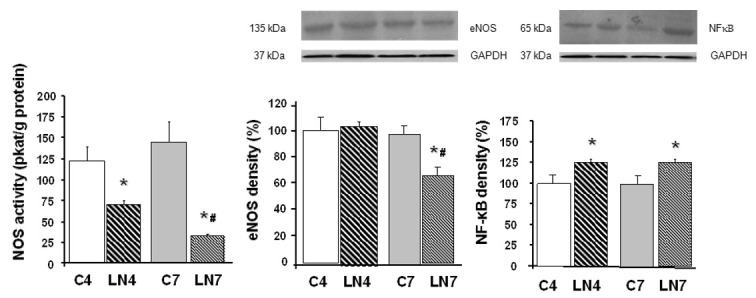
Effect of 4- and 7-week N^G^-nitro-L-arginine methyl ester (L-NAME) treatment on NO synthase (NOS) activity, endothelial NOS (eNOS) protein expression, and nuclear factor-kappaB (NF-κB, p65) protein expression in the cerebellar cortex. Group treated with L-NAME for 4 weeks (LN4) and age-matched controls (C4), group treated with L-NAME for 7 weeks (LN7) and age-matched controls (C7). * *p* < 0.05 compared to age-matched controls, # *p* < 0.05 compared to LN4.

Total NOS activity was decreased after 4 weeks of L-NAME treatment in all tissues investigated. However, when the treatment was prolonged to 7 weeks, NOS activity was increased in the aorta, heart, and kidney, while in the brain regions, it was markedly decreased (Figure 2, Figure 3, Figure 4, Figure 5, Figure 6 and Figure 7).

### 3.3. Western Blot Analysis

After 4 weeks of L-NAME treatment, eNOS protein expression in the aorta, heart, and kidney increased significantly and this increase was amplified after 7 weeks of treatment (Figure 2, Figure 3 and Figure 4). eNOS protein expression in the brainstem, cerebellum, and brain cortex remained unchanged after the 4-week-L-NAME treatment and prolongation of the treatment led to a significant decrease of eNOS expression in all brain regions examined (Figure 5, Figure 6 and Figure 7).

Protein expression of nNOS increased significantly only in the heart after the 7-week L-NAME treatment. There were no significant changes in the protein expression of the iNOS isoform (data not shown).

NF-κB (p65) protein expression increased in all tissues examined after 4 weeks of L-NAME treatment (Figure 2, Figure 3, Figure 4, Figure 5, Figure 6 and Figure 7) and prolongation of treatment decreased expression in the aorta, heart, and kidney (Figure 2, Figure 3 and Figure 4).

## 4. Discussion

To our knowledge, this is the first report comparing the different effects of chronic L-NAME- treatment on peripheral tissues and the brain. We found that prolonging the L-NAME treatment from 4 to 7 weeks increased NOS activity in the aorta, heart, and kidney, while in the brain regions, such as the brainstem, cerebellum, and brain cortex, the activity was significantly decreased. Increased expression of eNOS protein may be responsible for increased NOS activity in the peripheral tissues, while decreased expression of the same NOS isoform in the brainstem, cerebellum, and brain cortex led to a very pronounced decrease of NOS activity. Analysis of NF-κB (p65) protein expression also confirmed the regulatory role of this transcriptional factor for eNOS protein expression.

We assumed that upregulation of eNOS protein expression in peripheral tissues represents one of the counter-regulatory mechanisms activated to compensate decreased NO production and increased blood pressure. Similarly, Nava et al. [30] documented increased eNOS and nNOS expression in cardiac endothelial cells of spontaneously hypertensive rats as a result of the NO deficiency that accompanies spontaneous hypertension. Moreover, Llorens et al. [31] reported that the NO pathway was upregulated in the cardiovascular system and kidney both in aged normotensive and spontaneously hypertensive rats. Activation of NF-κB was suggested by Grumbach et al. [25] as one of the mechanisms responsible for eNOS upregulation. Numerous models of experimental hypertension, including L-NAME-induced hypertension, are characterized by increased levels of reactive oxygen species and NF-κB activation [17,18,19]. Grumbach et al. [25] hypothesized that under physiological conditions, the inhibitory effect of NO on NF-κB activation serves as a negative feedback mechanism to inhibit NF-κB activation and eNOS transcription. Since L-NAME prevented NF-κB (p50) nitrosylation and thus enabled translocation of NF-κB subunits to the nucleus, leading to increased eNOS mRNA expression, in the absence of NO, NF-κB stimulation and eNOS transcription were enhanced. Prolonged L-NAME treatment may consequently result in a significant increase of eNOS protein expression. Moreover, newly synthetized eNOS protein might be less sensitive to L-NAME due to the effect of increased tolerance. The more than doubled eNOS protein expression in the peripheral tissues (see Figure 2, Figure 3 and Figure 4) along with the reduced sensitivity to L-NAME might overtake the inhibitory effect of L-NAME on NOS and finally lead to paradoxically increased NOS activity in our experimental conditions. Taking into account that prolongation of L-NAME treatment further activated the systems responsible for increased blood pressure (RAAS, SNS, ROS), it is quite plausible that the adaptive mechanisms, including eNOS expression, are activated similarly to other models of experimental hypertension, e.g., spontaneous hypertension.

However, there is a question of when L-NAME-induced hypertension ceases to be L-NAME induced, and/or what is responsible for increased blood pressure after prolonged L-NAME treatment when the peripheral tissues are not involved. To address these questions, brain regions were analyzed. In contrast to the aorta, heart, and kidney, NOS inhibition was found to be increased in the brainstem, cerebellum, and brain cortex after prolongation of L-NAME treatment. Similarly, decreased NOS activity in the brain regions was documented by Majzunova et al. [32] and Kagiyama et al. [33] in NO-deficient and spontaneously hypertensive rats, respectively. It thus seems that decreased NOS activity in the brain may play an important role in the regulation of blood pressure in L-NAME-induced hypertension. Likewise, da Silva et al. [34] concluded that the central nervous system may have a dominant role in hypertension induced by chronic L-NAME treatment. They suggested, however, central melanocortin 3 and 4 receptors (MC3/4R) as the main factors contributing to this type of hypertension.

Regarding our study, concerns remain about the failure of NF-κB to induce upregulation of NOS in the brain regions where increased production of ROS and NF-κB activation were documented after L-NAME treatment in the central nervous system [29]. It was suggested that NO can stimulate DNA binding activity of NF-κB in some cell types while exerting an inhibitory effect in others [35,36,37]. NO and NO-generating compounds, such as S-nitroso-*n*-acetylpenicillamine (SNAP), increased NF-κB activity in human lymphocytes [35], while NO donors had an inhibitory effect on NF-κB activity in human coronary artery endothelial cells [36]. The study of Simpson and Morris [37] documented that exposure of rat striatum neurons to SNAP increased nuclear protein expression of both p50 and p65 subunits. In the same study, stimulation with NOR-3, an NO donor, increased the NF-κB DNA binding activity in the striatum of adult rats. It seems that NF-κB might be activated differentially in peripheral tissues, the central nervous system, and in the immune system. Furthermore, NO may induce opposite responses, i.e., activation or inhibition of NF-κB depending on NO concentration and type of the cells. Analysis of NF-κB (p65) protein expression under our experimental conditions virtually confirmed this hypothesis. Prolongation of L-NAME treatment to 7 weeks resulted in, along with an increased NO level, decreased NF-κB (p65) protein expression in peripheral tissues compared to the values of the 4-week treatment. On the other hand, there were no changes in NF-κB (p65) protein expression in the brain regions after 4 or 7 weeks of L-NAME treatment.

In conclusion, increased expression of eNOS may be responsible for increased NOS activity in the peripheral tissues studied after 7-week L-NAME treatment. Decreased expression of eNOS led, however, to a remarkable decrease of NOS activity in the brain regions. Since blood pressure increase persisted after 7 weeks of L-NAME treatment, we hypothesize that central regulation of blood pressure may contribute significantly to L-NAME-induced hypertension.

## Figures and Tables

**Table 1:** Body weight (BW), left ventricle weight (LVW), and relative left ventricle (LVW/BW) of Wistar rats treated with L-NAME (40 mg/kg/day) for 4 and 7 weeks and age-matched controls.

	Control 4	L-NAME 4	Control 7	L-NAME 7
BW (g)	330 ± 10	337 ± 15	341 ± 7	349 ± 10
LVW (mg)	439 ± 21	509 ± 23 *	465 ± 29	536 ± 20 *
LVW/BW (mg/g)	1.33 ± 0.04	1.51 ± 0.05 *	1.36 ± 0.07	1.54 ± 0.06 *

Data are means ± S.E.M., Significant differences: * *p* < 0.05 compared to age-matched controls.

## References

[1] Torres-Narváez J.C., Pérez-Torres I., Castrejón-Tellez V., Varela-López E., Oidor-Chan V.H., Guarner-Lans V., Vargas-González Á., Memije R.M., Flores-Chávez P., Cervantes-Yañez E.Z. (2019). The Role of the Activation of the TRPV1 Receptor and of Nitric Oxide in Changes in Endothelial and Cardiac Function and Biomarker Levels in Hypertensive Rats. Int. J. Environ. Res. Public Health.

[2] Sonoda K., Ohtake K., Uchida H., Ito J., Uchida M., Natsume H., Tamada H., Kobayashi J. (2017). Dietary nitrite supplementation attenuates cardiac remodeling in l -NAME-induced hypertensive rats. Nitric Oxide.

[3] Babál P., Pechánová O., Bernátová I., Stvrtina S. (1997). Chronic inhibition of NO synthesis produces myocardial fibrosis and arterial media hyperplasia. Histol. Histopathol..

[4] Adedara I.A., Alake S.E., Olajide L.O., Adeyemo M.O., Ajibade T.O., Farombi E.O. (2018). Taurine Ameliorates Thyroid Hypofunction and Renal Injury in L-NAME-Induced Hypertensive Rats. Drug Res..

[5] Guzmán-Hernández E.A., Villalobos-Molina R., Sánchez-Mendoza M.A., Del Valle-Mondragón L., Pastelín-Hernández G., Ibarra-Barajasm M. (2015). Early co-expression of cyclooxygenase-2 and renin in the rat kidney cortex contributes to the development of N(G)-nitro-L-arginine methyl ester induced hypertension. Can. J. Physiol. Pharmacol..

[6] Pechanova O., Matuskova J., Capikova D., Jendekova L., Paulis L., Simko F. (2006). Effect of spironolactone and captopril on nitric oxide and S-nitrosothiol formation in kidney of L-NAME-treated rats. Kidney Int..

[7] Aekthammarat D., Pannangpetch P., Tangsucharit P. (2019). Moringa oleifera leaf extract lowers high blood pressure by alleviating vascular dysfunction and decreasing oxidative stress in L-NAME hypertensive rats. Phytomedicine.

[8] Holecyova A., Torok J., Bernatova I., Pechanova O. (1996). Restriction of nitric oxide rather then elevated blood pressure is responsible for alterations of vascular responses in nitric oxide deficient hypertension. Physiol. Res..

[9] Yadav V.R., Teng B., Mustafa S.J. (2019). Enhanced A1 adenosine receptor-induced vascular contractions in mesenteric artery and aorta of in L-NAME mouse model of hypertension. Eur. J. Pharmacol..

[10] Ignarro L.J. (2002). Nitric oxide as a unique signaling molecule in the vascular system: A historical overview. J. Physiol. Pharmacol..

[11] Simko F., Baka T., Poglitsch M., Bednarova K.R., Aziriova S., Krajcirovicova K., Zorad S., Adamcova M., Paulis L. (2018). Effect of Ivabradine on a Hypertensive Heart and the Renin-Angiotensin-Aldosterone System in L-NAME-Induced Hypertension. Int. J. Mol. Sci..

[12] Villarejo A., Prieto I., Segarra A., Banegas I., Wangensteen R., Vives F., De Gasparo M., Ramírez-Sánchez M. (2014). Relationship of Angiotensinase and Vasopressinase Activities Between Hypothalamus, Heart, and Plasma in L-NAME-Treated WKY and SHR. Horm. Metab. Res..

[13] Giani J.F., Janjulia T., Kamat N., Seth D.M., Blackwell W.-L.B., Shah K.H., Shen X.Z., Fuchs S., Delpire E., Toblli J.E. (2014). Renal Angiotensin-Converting Enzyme Is Essential for the Hypertension Induced by Nitric Oxide Synthesis Inhibition. J. Am. Soc. Nephrol..

[14] Cavalcante G.L., Ferreira F.N., Da Silva M.T.B., Soriano R.N., Filho A.L.M.M., Arcanjo D.D.R., Sabino J.P.J. (2020). Acetylcholinesterase inhibition prevents alterations in cardiovascular autonomic control and gastric motility in L-NAME-induced hypertensive rats. Life Sci..

[15] Chaswal M., Das S., Prasad J., Katyal A., Fahim M. (2015). Chemical Sympathectomy Restores Baroreceptor-Heart Rate Reflex and Heart Rate Variability in Rats with Chronic Nitric Oxide Deficiency. Physiol. Res..

[16] Colonna V.D.G., Fioretti S., Rigamonti A., Bonomo S., Manfredi B., Muller E.E., Berti F., Rossoni G. (2006). Angiotensin II type 1 receptor antagonism improves endothelial vasodilator function in L-NAME-induced hypertensive rats by a kinin-dependent mechanism. J. Hypertens..

[17] Kanthlal S.K., Joseph J., Paul B. (2020). Antioxidant and vasorelaxant effects of aqueous extract of large cardamom in L-NAME induced hypertensive rats. Clin. Exp. Hypertens..

[18] Rincón J., Correia D., Arcaya J., Finol E., Fernández A., Pérez M., Yaguas K., Talavera E., Chávez M., Summer R. (2015). Role of Angiotensin II type 1 receptor on renal NAD(P)H oxidase, oxidative stress and inflammation in nitric oxide inhibition induced-hypertension. Life Sci..

[19] Pechanova O., Varga Z.V., Cebova M., Giricz Z., Pacher P., Ferdinandy P. (2014). Cardiac NO signalling in the metabolic syndrome. Br. J. Pharmacol..

[20] Leo M.D., Kandasamy K., Subramani J., Tandan S.K., Kumar D. (2015). Involvement of inducible nitric oxide synthase and dimethyl arginine dimethylaminohydrolase in Nω-Nitro-L-arginine methyl ester (L-NAME)-induced hypertension. Cardiovasc. Pathol..

[21] Sollinger D., Eißler R., Lorenz S., Strand S., Chmielewski S., Aoqui C., Schmaderer C., Bluyssen H., Zicha J., Witzke O. (2014). Damage-associated molecular pattern activated Toll-like receptor 4 signalling modulates blood pressure in l-NAME-induced hypertension. Cardiovasc. Res..

[22] Kosutova M., Pechanova O., Barta A., Franova S., Cebova M. (2019). Different adaptive NO-dependent Mechanisms in Normal and Hypertensive Conditions. Molecules.

[23] Silambarasan T., Manivannan J., Priya M.K., Suganya N., Chatterjee S., Raja B. (2014). Sinapic Acid Prevents Hypertension and Cardiovascular Remodeling in Pharmacological Model of Nitric Oxide Inhibited Rats. PLoS ONE.

[24] Berkban T., Boonprom P., Bunbupha S., Welbat J.U., Kukongviriyapan U., Kukongviriyapan V., Pakdeechote P., Prachaney P. (2015). Ellagic Acid Prevents L-NAME-Induced Hypertension via Restoration of eNOS and p47phox Expression in Rats. Nutrition.

[25] Grumbach I.M., Chen W., Mertens S., Harrison D. (2005). A negative feedback mechanism involving nitric oxide and nuclear factor kappa-B modulates endothelial nitric oxide synthase transcription. J. Mol. Cell. Cardiol..

[26] Bruno A.S., Lopes P.D.D., De Oliveira K.C.M., De Oliveira A.K., Cau S.B.A. (2019). Vascular Inflammation in Hypertension: Targeting Lipid Mediators Unbalance and Nitrosative Stress. Curr. Hypertens. Rev..

[27] Sun Y., Carretero O.A., Xu J., Rhaleb N.-E., Yang J.J., Pagano P.J., Yang X.-P. (2009). Deletion of Inducible Nitric Oxide Synthase Provides Cardioprotection in Mice With 2-Kidney, 1-Clip Hypertension. Hypertension.

[28] Kitamoto S., Egashira K., Kataoka C., Koyanagi M., Katoh M., Shimokawa H., Morishita R., Kaneda Y., Sueishi K., Takeshita A. (2000). Increased activity of nuclear factor-kappaB participates in cardiovascular remodeling induced by chronic inhibition of nitric oxide synthesis in rats. Circulation.

[29] Pechanova O., Jendeková L., Kojšová S., Jagla F. (2006). Possible role of nitric oxide in the locomotor activity of hypertensive rats. Behav. Brain Res..

[30] Nava E., Noll G., Lüscher T.F. (1995). Increased Activity of Constitutive Nitric Oxide Synthase in Cardiac Endothelium in Spontaneous Hypertension. Circulation.

[31] Llorens S., Fernández A.P., Nava E. (2007). Cardiovascular and renal alterations on the nitric oxide pathway in spontaneous hypertension and ageing. Clin. Hemorheol. Microcirc..

[32] Majzúnová M., Pakanová Z., Kvasnička P., Bališ P., Čačányiová S., Dovinová I. (2017). Age-dependent redox status in the brain stem of NO-deficient hypertensive rats. J. Biomed. Sci..

[33] Kagiyama S., Tsuchihashi T., Abe I., Fujishima M. (1998). Enhanced Depressor Response to Nitric Oxide in the Rostral Ventrolateral Medulla of Spontaneously Hypertensive Rats. Hypertension.

[34] da Silva A.A., do Carmo J.M., Dubinion J.H., Bassi M., Mokhtarpouriani K., Hamza S.M., Hall J.E. (2015). Chronic central nervous system MC3/4R blockade attenuates hypertension induced by nitric oxide synthase inhibition but not by angiotensin II infusion. Hypertension.

[35] Lander H.M., Sehajpal P., Levine D.M., Novogrodsky A. (1993). Activation of human peripheral blood mononuclear cells by nitric oxide-generating compounds. J. Immunol..

[36] Zhen J., Lu H., Wang X.Q., Vaziri N.D., Zhou X.J. (2008). Upregulation of Endothelial and Inducible Nitric Oxide Synthase Expression by Reactive Oxygen Species. Am. J. Hypertens..

[37] Simpson C.S., Morris B.J. (1999). Activation of nuclear factor kappaB by nitric oxide in rat striatal neurones: Differential inhibition of the p50 and p65 subunits by dexamethasone. J. Neurochem..

